# The Inhibitory Role of Hydrogen Sulfide in UII-Induced Cardiovascular Effects and the Underlying Signaling Pathways

**DOI:** 10.3390/antiox11112253

**Published:** 2022-11-15

**Authors:** Na-Na Zhang, Hai-Yan Xu, Xiao-Ni Liu, Yi-Fan Chen, Chun-Mei Xia, Xing-Zhong Wu, Ning Lu

**Affiliations:** 1 Department of Physiology and Pathophysiology, School of Basic Medical Sciences, Fudan University, Shanghai 200032, China; 2 Department of Urology, Fudan Institute of Urology, Huashan Hospital, Fudan University, Shanghai 200040, China; 3 Department of Neurology, Huashan Hospital, Fudan University, Shanghai 200040, China; 4 Department of Biochemistry and Molecular Biology, School of Basic Medical Sciences, Fudan University, Shanghai 200032, China

**Keywords:** urotensin II, H_2_S, hypertension, ROS, ERK1/2, p38MAPK, Gα

## Abstract

Urotensin II (UII) could increase blood pressure and heart rate via increased central reactive oxygen species (ROS) levels. We reported previously that hydrogen sulfide (H_2_S) exerts an antihypertensive effect by suppressing ROS production. The aim of the current study is to further examine the effects of endogenous and exogenous H_2_S on UII-induced cardiovascular effects by using an integrated physiology approach. We also use cell culture and molecular biological techniques to explore the inhibitory role of H_2_S on UII-induced cardiovascular effects. In this study, we found that cystathionine-β-synthase (CBS), the main H_2_S synthesizing enzyme in CNS, was expressed in neuronal cells of the rostral ventrolateral medulla (RVLM) area. Cellular distribution of CBS and urotensin II receptor (UT) in SH-SY5Y cells that are confirmed as glutamatergic were identified by immunofluorescent and Western blots assay. In Sprague–Dawley rats, administration of UII into the RVLM resulted in an increase in mean arterial pressure (MAP), heart rate (HR), ROS production, nicotinamide adenine dinucleotide phosphate (NADPH) oxidase activity, and phosphorylation of p47^phox^, extracellular signal-regulated protein kinase (ERK)1/2 and p38MAPK, but not stress-activated protein kinase/Jun N-terminal kinase (SAPK/JNK). These effects of UII were attenuated by application into the RVLM of endogenous (L-cysteine, SAM) or exogenous (NaHS) H_2_S. These results were confirmed in SH-SY5Y cells. UII-induced cardiovascular effects were also significantly abolished by pretreatment with microinjection of Tempol, Apocynin, SB203580, or PD98059 into the RVLM. Preincubated SH-SY5Y cells with Apocynin before administration of UII followed by Western blots assay showed that ROS is in the upstream of p38MAPK/ERK1/2. Ga_o_ activation assay in SH-SY5Y cells suggested that H_2_S may exert an inhibitory role on UII-induced cardiovascular effects by inhibiting the activity of Gα_o_. These results suggest that both endogenous and exogenous H_2_S attenuate UII-induced cardiovascular effects via Gα_o_-ROS-p38MAPK/ERK1/2 pathway.

## 1. Introduction

Urotensin II (UII), a cyclic neuropeptide, was originally found in teleost fish and later characterized in mammals [1]. It exerts a vasoactive effect through binding and activating an ancestral receptor, a G-protein coupled receptor, termed the UT receptor. UII and UT are widely expressed in the cardiovascular system, various peripheral organs, and the central nervous system (CNS). Our previous work [2] showed that microinjection of UII in the rostral ventrolateral medulla (RVLM) of spontaneously hypertensive rats (SHR) could increase blood pressure and heart rate through decreased central reactive oxygen species (ROS) and inhibition of NADPH oxidase.

Oxidative stress is a significant feature of hypertension [3]. A growing body of evidence indicates that ROS is an important mediator of sympathetic activation. Angiotensin II and urotensin II (UII) [4] could increase blood pressure and heart rate (HR) via an increase in the central ROS levels. The rostral ventrolateral medulla (RVLM) is the key region of cardiovascular center, which contains sympathoexcitatory neurons. It is vital for oxidative stress in the mechanism of hypertension because of its pivotal role in regulating vascular tone and HR [5]. We reported previously [6] that increased mean arterial pressure (MAP), HR, and reactive oxygen species (ROS) production, derived from NADPH oxidase, were demonstrated in SHR rats following microinjection of UII into the RVLM. Chan et al. [7] reported angiotensin II-induced cellular events in the RVLM through NADPH oxidase-derived O_2_^−^, activated ERK1/2 and p38MAPK. Thus, decreased ROS generation may be an important target of antihypertension therapy.

Hydrogen sulfide (H_2_S), a gasotransmitter, was reported to have various physiological effects on the cardiovascular system, nervous system, and metabolism, such as antioxygen, antihypertensive, vascular relaxation, and maintaining blood pressure homeostasis [8,9]. H_2_S also impacts transcriptional factors such as NF-Kappa B and modulates T cell inflammation and immunosuppression [10]. H_2_S is endogenously synthesized by cystathionine-β-synthase (CBS), cystathionine-γ-lyase (CSE), and 3-mercaptopyruvate sulfurtransferase (3-MST), with 3-MST and CBS expressed in the CNS, while the expression of CBS is predominant. Chronic intracerebroventricular infusion of NaHS, the donor of H_2_S, can alleviate angiotensin II-induced hypertension [11]. Our previous study indicated that H_2_S could reduce MAP and HR and the level of ROS in the RVLM of SHR rats [2]. More importantly, H_2_S can decrease ROS generation and NADPH oxidase activity via inhibition of ERK1/2. However, the mechanisms underlying the central cardiovascular effects of H_2_S remain largely unknown.

Based on the above research, UII exerts its pressor effect through coupling to the UT receptor, a G-protein coupled receptor. In the pathological conditions of hypertension, G-protein coupled receptor involves cross talk between various signaling pathways, including mitogen-activated protein kinases (MAPK) [12]. It has been shown that the UT receptor couples to Gα_i/o_ proteins leading to an increase in phosphorylation of extracellular signal-regulated kinase 1/2 (ERK1/2) [13]. On the other hand, G-protein coupled receptor agonist is also involved in the activation of nicotinamide adenine dinucleotide phosphate (NADPH) oxidase [14].

In the present study, we tested the hypothesis that H_2_S may exert an antihypertensive effect induced by UII by decreasing ROS production. Furthermore, we identified that the MAPK signaling pathway and Gα_i/o_ protein might also be involved in the mechanisms of the antihypertensive effect of H_2_S. Our study may lead to a potential novel therapeutic strategy for hypertension.

## 2. Materials and Methods

### 2.1. Animals

Adult male Sprague–Dawley rats weighing 280–310 g were purchased from Shanghai SLAC laboratory animal company. All experiments were approved by the animal experimental Ethics Committee of Fudan University (20210302-038).

### 2.2. Cell Culture

SH-SY5Y cells were cultured with 10% fetal bovine serum (FBS) and 1% penicillin–streptomycin in Dulbecco’s Modified Eagle’s Medium (DMEM) under 95% air and 5% carbon dioxide (CO_2_) at 37 °C.

### 2.3. Immunofluorescence Staining

Animals were anesthetized deeply with a mixture of chloralose (35 mg/kg) and urethane (700 mg/kg) and perfused transcardially with saline (150 mL) followed by 4% paraformaldehyde (250 mL) in 0.1 M sodium phosphate buffer (0.1 M PBS; pH 7.4). Brains were rapidly removed and post-fixed in the same fixative at 4 °C overnight, followed by transfer into 20% and 30% sucrose sequentially in 0.1 M PB for at least 3 days. According to Paxinos and Watson’s atlas, coronal medullary sections at 30 μm thick were cut with a microtome (Leica, Deer Park, IL, USA) 1.5–1.7 mm rostral to the obex. For cells, 4% paraformaldehyde was performed for 30 min after the cells were treated with UII or NaHS. After washing in 0.01 M phosphate-buffered saline (0.01 M PBS; pH 7.4) and absorption in 2% BSA, and 0.2% Triton X-100 in PBS, the sections or the cells were incubated with primary antibodies at 4 °C overnight. Anti-MAP-2 and anti-CBS antibodies were purchased from Abcam (Cambridge, UK). Anti-glutamate antibody was purchased from Sigma-Aldrich. Sections or the cells were then washed in PBS and incubated with fluorescent secondary antibodies for 2 h. The sections or the cells were observed using confocal microscopy (Zeiss LSM510, Jena, Germany).

### 2.4. Intracerebroventricular Infusion

After the rats were anesthetized, a hole (1.2–1.4 mm lateral to the midline and 0.8–1.0 mm posterior to bregma) was made in the skull. A stainless steel guide cannula (10 mm, 22 gauges) and a stainless steel injector, which was introduced through the guide cannula, were inserted into the hole. A total of 10 μL was delivered over 10 min into the ICV.

### 2.5. Microinjection into the RVLM

Animals were anesthetized with a mixture of chloralose (35 mg/kg) and urethane (700 mg/kg) and intubated to facilitate ventilation. Blood pressure and heart rate were recorded by inserting a cannula into the left femoral artery. Body temperature was maintained with a temperature-controlled table between 37 °C and 37.5 °C. Rats were then mounted in a stereotaxic frame, and microinjection was carried out with a micropipette tip (external tip diameter 0.4 mm). A total of 0.1 μL was delivered over 1 min into the RVLM (1.8 to 2.1 mm lateral to midline, 2.6 to 3.3 mm caudal to interaural line, and 0.3 to 0.9 mm from the ventral surface). The injection sites were confirmed by histological examination. The test agents, including L-cysteine (a CBS substrate), S-adenosyl-l-methionine (SAM, a CBS agonist), HA, Tempol, Apocynin, SB203580, PD98059, and NaHS, were purchased from Sigma Aldrich (St. Louis, MO, USA). UII was synthesized in GL biochemistry. BIM23127 was purchased from Tocris (Bristol, UK). Microinjection of artificial cerebral spinal fluid (aCSF) was used as vehicle control and volume.

### 2.6. ROS

As described previously [2], superoxide production in the ventrolateral medulla was detected using a lucigenin-enhanced chemiluminescence assay. The ventrolateral medulla was administrated with different agents, then removed and homogenized in PBS containing 0.01 mM EDTA. After centrifuging, the supernatant was obtained and added to a buffer containing lucigenin (5 μmol/L) for O_2_^−^ measurement. O_2_^−^ production was expressed as the mean light unit per mg protein.

Superoxide production in SH-SY5Y cells was detected using ROS fluorescent probe, dihydroethidium (DHE, Sigma-Aldrich, St. Louis, MO, USA). Cells were treated with various agents, followed by incubation with 2 μmol/L DHE for 30 min. ROS production was analyzed by measuring the absorbance at 480 nm and 590 nm.

### 2.7. Measurement of NADPH Oxidase Activity

In the ventrolateral medulla, NADPH oxidase activity was analyzed by a luminescence assay. The assay was performed in PBS buffer containing EGTA (0.01 M/L) and lucigenin (5 μmol/L) as the electron acceptor and NADPH (100 mM/L) as the substrate. Background counts were recorded after dark adaptation, followed by the addition of a tissue homogenate (1 μL protein sample). The chemiluminescence value was recorded at 1 min intervals for 30 min. After the addition of NADPH, O_2_^−^ production in the incubation medium was measured.

### 2.8. Western Blotting

As described previously, tissue homogenates or cell lysates were subjected to Western blot analysis. BCA protein assay reagent (Pierce, Appleton, WI, USA) was used to assay protein content. Protein was resolved by SDS-PAGE in equal amounts and transferred to the PVDF membrane. Blots were incubated with primary antibody followed by horseradish peroxidase-conjugated secondary antibody. Anti-CBS antibodies were purchased from Abcam (Waltham, MA, USA). Anti-UT antibody was purchased from Santa Cruz Biotechnology (Dallas, TX, USA). Anti-total p47^phox^ antibody was purchased from Sigma-Aldrich (St. Louis, MO, USA). Anti-total or phosphorylated ERK1/2, p38MAPK, or JNK and phosphorylated p-p47^phox^ antibodies were purchased from Cell Signaling (Danvers, MA, USA). Protein bands were visualized by ECL (GE Healthcare, Chicago, IL, USA). The intensity (area × density) of the detected protein on Western blots was quantified by Image J software and was expressed as the ratio to the total protein amount.

### 2.9. Cell Viability Assay

Cell Counting Kit-8 (CCK-8, obtained from Dojindo, Munich, Germany) was used to determine cell viability. The SH-SY5Y cells were cultured in 96-well plates at 37 °C in a 5% CO_2_ incubator. Cells were treated with different concentrations of UII and NaHS at different times. Then 10 μL CCK-8 solution was added to each well of plates then incubated for 2 h at 37 °C, 5% CO_2_. The cell viability was analyzed by measuring the absorbance at 450 nm. 

### 2.10. Gα_o_ Activation Assay

The activity of Ga_o_ was determined using Gα_o_ Activation Assay Kit (NewEast Biosciences, Wuhan, China) according to the manufacturer’s instructions.

### 2.11. Statistical Analysis

Data are expressed as mean ± SEM. Differences between groups were compared using one-way ANOVA associated with Tukey-Kramer post hoc comparison or the unpaired Student’s *t*-test for parametric data. Statistical significance was assumed when *p*-values below 0.05.

## 3. Results

### 3.1. Expression of CBS and UT

**Figure 1:**
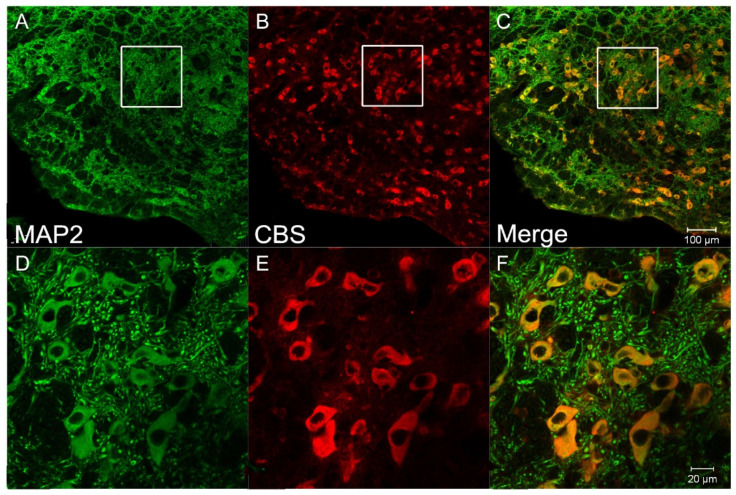
CBS expressed in the cytoplasm of RVLM neurons. Confocal images showed that CBS immunoreactivity (showed in red, (**B**,**E**)) is co-localized with a neuronal marker (MAP2 showed in green, (**A**,**D**)). Figure (**C**,**F**) represents the overlay image. All nuclei are stained with DAPI. Bars = 100 µm (**A**–**C**). Bars = 20 µm (**D**–**F**).

**Figure 2:**
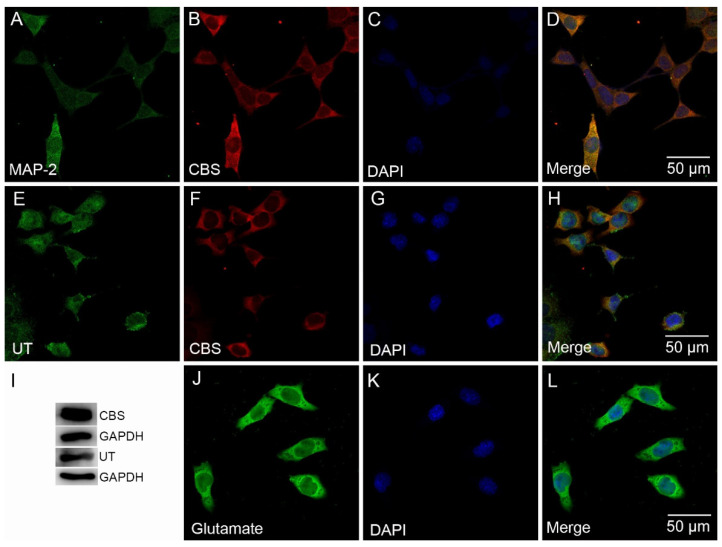
CBS and UT expression in SH-SY5Y cells. Fluorescence micrograph of cells stained with anti-MAP-2 antibody (showed in green, (**A**)) and anti-CBS antibody (showed in red, (**B**)). Fluorescence micrograph of cells stained with anti-UT antibody (showed in green, (**E**)) and anti-CBS antibody (showed in red, (**F**)). CBS expressed in the cytoplasm, and UT is mainly expressed in the cell membrane. All nuclei are stained with DAPI (**C**,**G**). Figure (**D**,**H**) represent the overlay image. Western blots analysis showed UT and CBS expression (**I**). Confocal images showed a fluorescence micrograph of cells stained with anti-glutamate antibody (showed in green, (**J**)). All nuclei are stained with DAPI (**K**). Figure (**L**) represent the overlay image. Bars = 50 µm.

To clarify the expression of H_2_S synthesizing enzymes and UT, we performed an immunofluorescent stain coupled with laser confocal microscopy on the RVLM area of SD rats and SH-SY5Y cells. The results confirmed that CBS, the main H_2_S synthesizing enzyme in CNS, was expressed in neuronal cells of the RVLM area (Figure 1) and SH-SY5Y cells (Figure 2). Cellular distribution of UT was identified in SH-SY5Y cells. The expression of CBS and UT in SH-SY5Y cells was further clarified by Western blots assay. We also found that SH-SY5Y cells are glutamatergic (Figure 2).

### 3.2. Effects of H_2_S on UII-Induced MAP and HR

**Figure 3:**
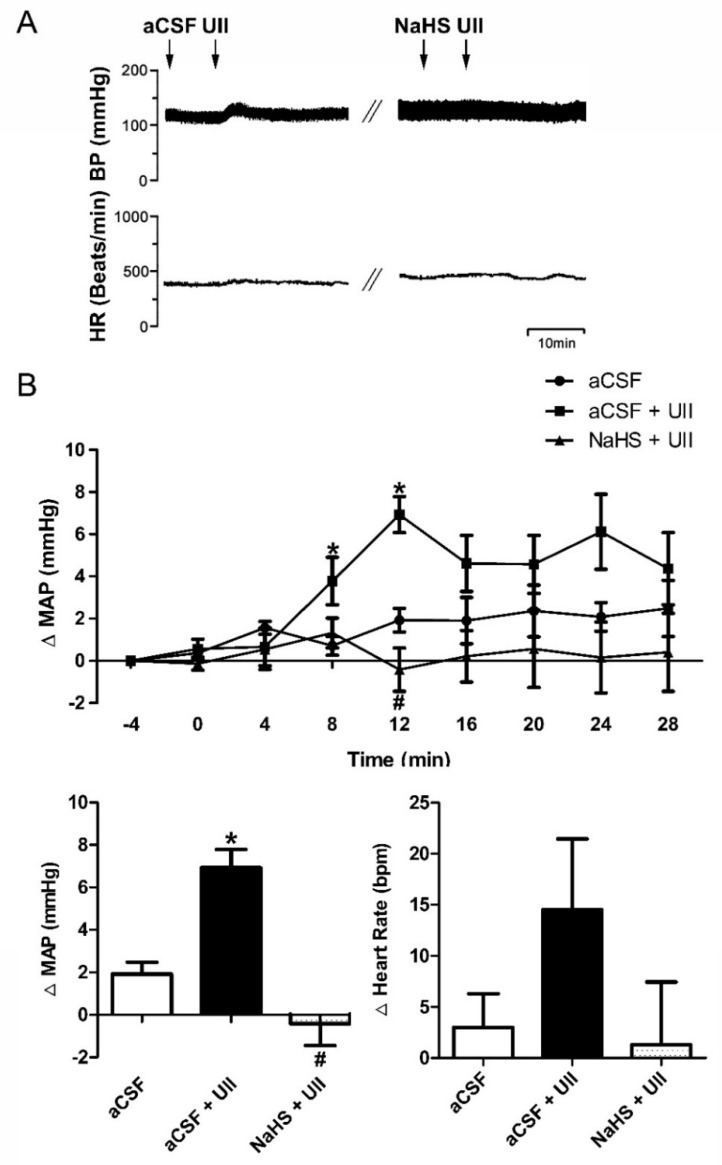
Microinjection of NaHS (400 pmol/0.1 μL) into RVLM decreased the induced upregulation of MAP and HR by UII in SD rats. Typical MAP and HR traces in response to the microinjection (**A**). Time courses of MAP in response to microinjection of aCSF (*n* = 6), aCSF + UII (*n* = 9), or NaHS + UII (*n* = 4) and the maximal changes detected during the response (**B**). * *p* < 0.05 vs. aCSF group; ^#^ *p* < 0.05 vs. aCSF + UII group.

**Figure 4:**
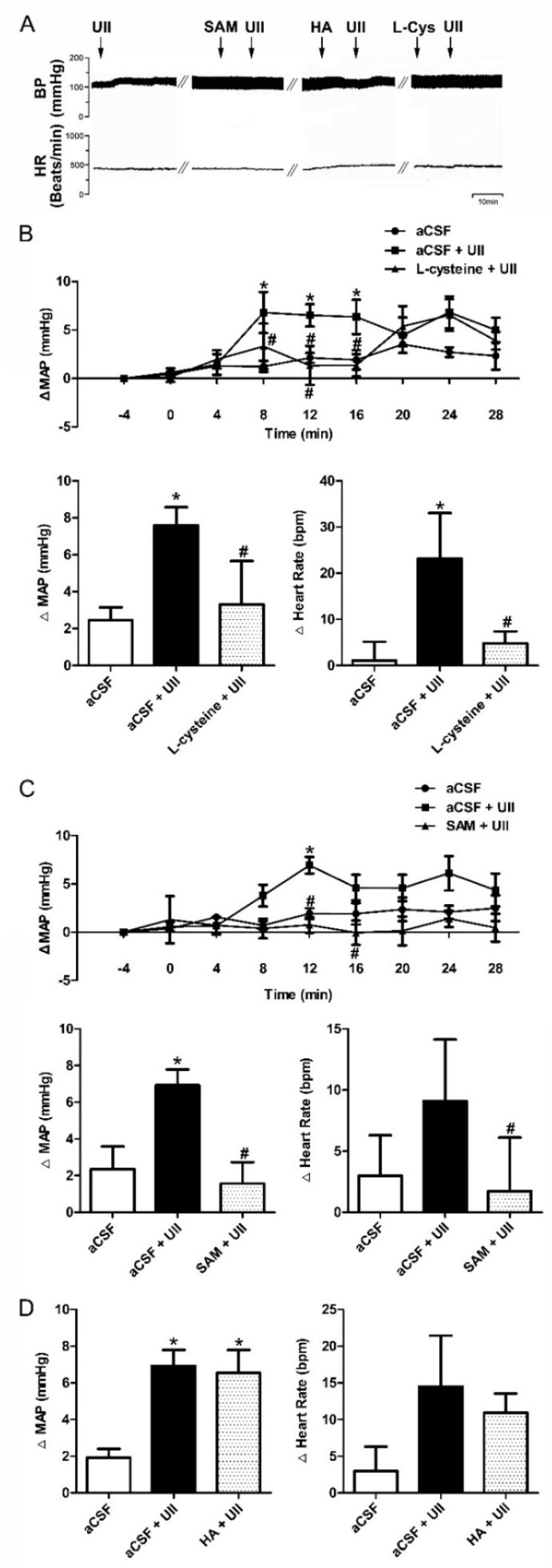
Effects of endogenous H_2_S on UII−induced MAP and HR in SD rats. Typical MAP and HR traces in response to the microinjection in SD rats (**A**). Time courses of MAP in response to microinjections of aCSF (*n* = 6), aCSF + UII (*n* = 6), or L−cysteine (0.034 nmol/0.1 μL) + UII (*n* = 6) and the maximal changes detected during the response (**B**). Time courses of MAP in response to microinjections of aCSF (*n* = 6), aCSF + UII (*n* = 9), or SAM (0.01 nmol/0.1 μL) + UII (*n* = 6) and the maximal changes detected during the response (**C**). Maximal changes detected during the response to microinjections of aCSF (*n* = 7), aCSF + UII (*n* = 9), or HA (3 nmol/0.1 μL) + UII (*n* = 5) (**D**). * *p* < 0.05 vs. aCSF group; ^#^ *p* < 0.05 vs. aCSF + UII group.

We first performed microinjection of UII (0.5 nmol/0.1 μL) into the RVLM of SD rats. The results showed that the MAP and HR of SD rats were increased within 5 min, while coadministration of NaHS (400 pmol/0.1 μL), an exogenous H_2_S donor, discernibly attenuated the effect induced by UII (Figure 3). Furthermore, increase in the production of endogenous H_2_S through pretreatment with microinjection of L-cysteine (a CBS substrate, 0.034 nmol/0.1 μL, Figure 4B) and SAM (a CBS agonist, 0.01 nmol/0.1 μL, Figure 4C), but not HA (a CBS antagonist, 3 nmol/0.1 μL, Figure 4D), into the RVLM decreased the induced upregulation of MAP and HR by UII (0.5 nmol/0.1 μL).

### 3.3. Effects of H_2_S on UII-Induced O_2_^−^ Production and NADPH Oxidase Activity

We reported previously [2] that microinjection of H_2_S into the RVLM of SHR rats decreased the level of superoxide anion (O_2_^−^) and NADPH oxidase activity. To further identify the underlying mechanism of the inhibitory effect of H_2_S on UII-induced cardiovascular effect, ROS production and NADPH oxidase activity were detected.

**Figure 5:**
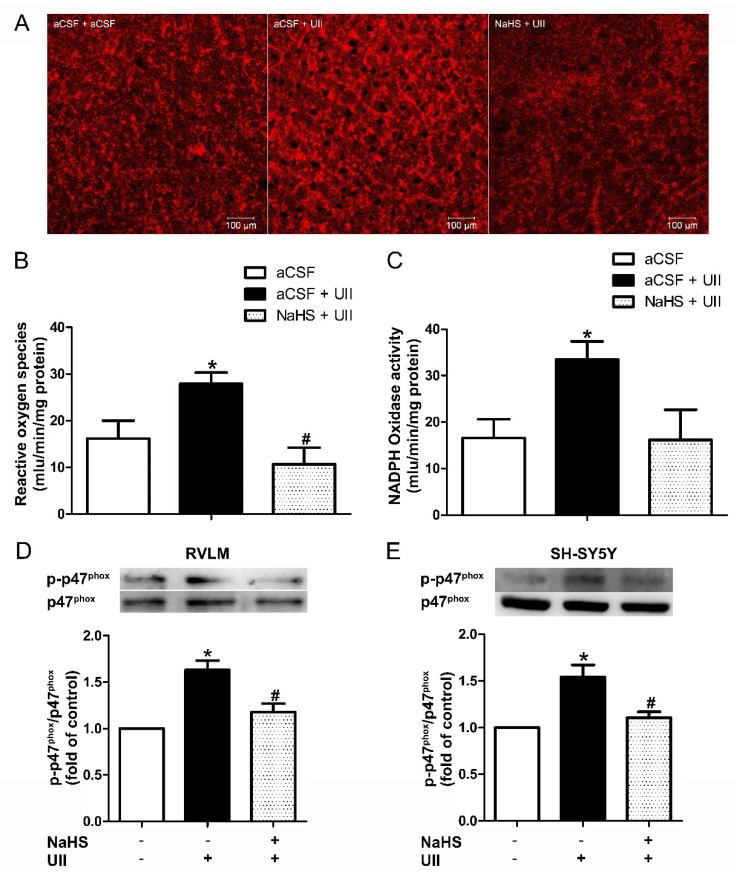
Microinjection of NaHS (400 pmol/0.1 μL) into the RVLM suppressed UII−induced superoxide production rise in SD rats. Representative images of reactive oxygen species (ROS) levels as assessed by dihydroethidium (DHE) staining in brain slices from RVLM (**A**). Tissue levels of superoxide anion (**B**) and NADPH oxidase activity (**C**) after microinjection of aCSF (*n* = 6, 4), aCSF + UII (*n* = 6, 4), or NaHS + UII (*n* = 6, 4). The change in p−p47^phox^ after microinjection of aCSF (*n* = 6), aCSF + UII (*n* = 6), or NaHS + UII (*n* = 6) into the RVLM of SD rats (**D**). The change in p−p47^phox^ after pretreated with NaHS (50 μmol/L) for 30 min before UII (10^−8^ mol/L) was used (**E**). * *p* < 0.05 vs. aCSF group; ^#^ *p* < 0.05 vs. aCSF + UII group.

In vivo, the ROS production was detected after RVLM was administrated with UII (0.5 nmol/0.1 μL). The results revealed that the superoxide anions (O_2_^−^) production in the RVLM was significantly enhanced upon UII administration, and this effect of UII was blocked by pretreatment with NaHS (400 pmol/0.1 μL) into the RVLM (Figure 5A,B).

NADPH oxidase activity in the RVLM was dramatically increased by UII, and this effect was diminished by pretreatment with microinjection of NaHS (400 pmol/0.1 μL) into the RVLM (Figure 5C). Phosphorylation of the p47^phox^ subunit is an important step for the activation of NADPH oxidase. Pretreated the RVLM area (Figure 5D) or SH-SY5Y cells (Figure 5E) with UII activated the p47^phox^ subunit of NADPH oxidase. The UII-induced increase in phosphorylation of the p47^phox^ subunit was significantly decreased by coadministration of NaHS, indicating that NaHS decreases the production of superoxide via suppression of the activity of NADPH oxidase.

**Figure 6:**
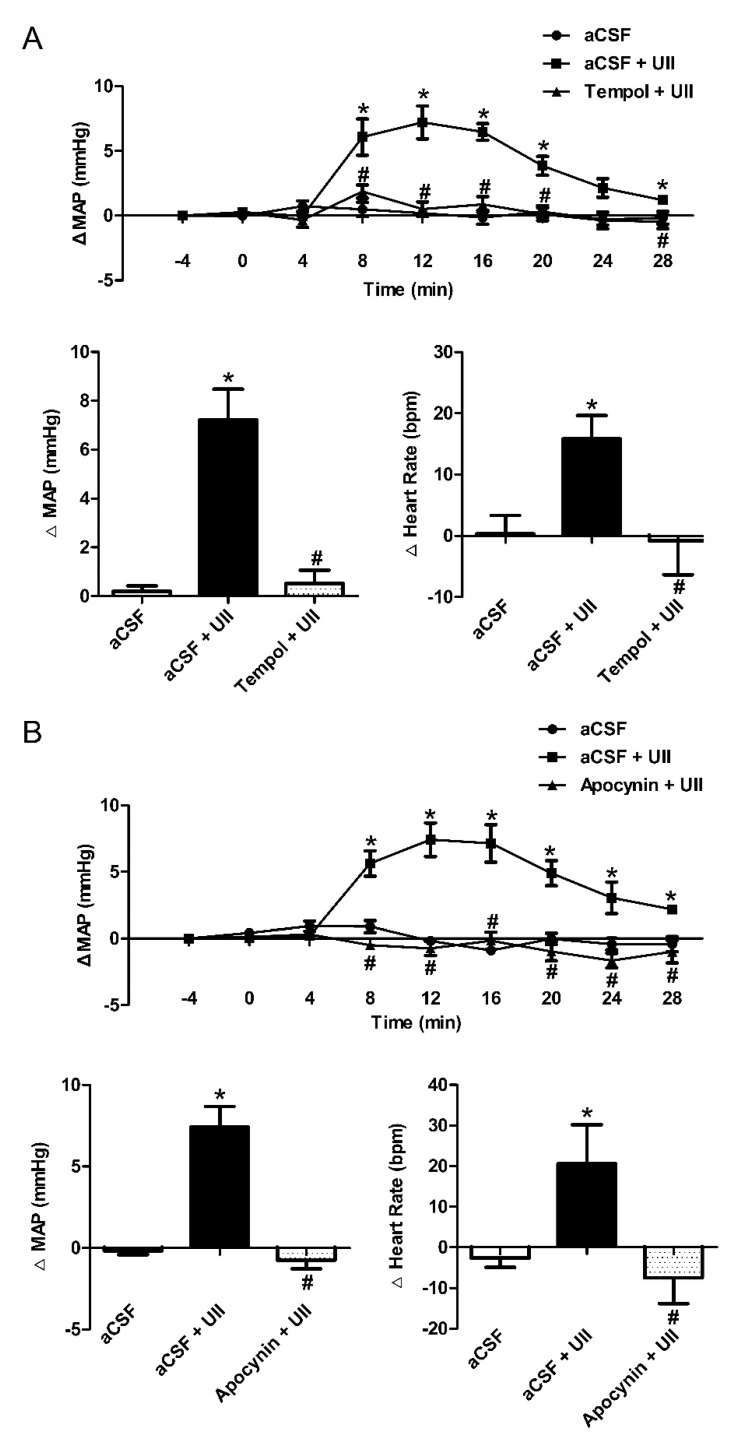
Microinjection of Tempol (a superoxide dismutase mimic) or Apocynin (NADPH oxidase inhibitor) into the RVLM decreased MAP and HR in SD rats. Time course of MAP in response to microinjection of aCSF (*n* = 6), aCSF + UII (*n* = 6), or Tempol (50 nmol/0.1 μL, (**A**))/Apocynin (5 nmol/0.1 μL, (**B**)) + UII (*n* = 6, 6) and the maximal changes detected during the response. * *p* < 0.05 vs. aCSF group; ^#^ *p* < 0.05 vs. aCSF + UII group.

To further confirm the effects of ROS on UII-induced hypertension, we performed the microinjection into the RVLM area of SD rats. Microinjection of UII (0.5 nmol/0.1 μL) into the RVLM evoked an appreciable increase in MAP and HR that lasted for 10 min. This pressor response was significantly inhibited by pretreatment with microinjection of Tempol (a superoxide dismutase mimic, 50 nmol/0.1 μL, Figure 6A) or Apocynin (NADPH oxidase inhibitor, 5 nmol/0.1 μL, Figure 6B) into the RVLM. However, UII or NaHS had no effect on SOD (data not shown).

**Figure 7:**
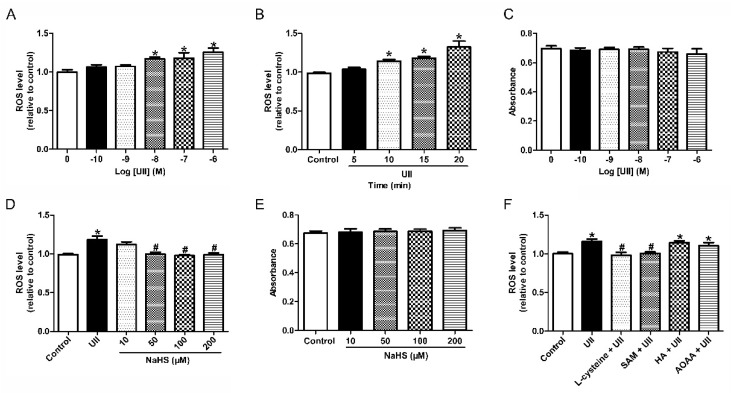
Effect of H_2_S on UII-induced increase in ROS level in SH-SY5Y cells. Bar graphs summarizing ROS level after treatment with various concentrations of UII (10^−6^, 10^−7^, 10^−8^, 10^−9^ or 10^−10^ mol/L, (**A**)). ROS levels were determined after pretreated SH-SY5Y cells with UII (10^−8^ mol/L) for different times (5, 10, 15, and 20 min, (**B**)) or various concentrations of NaHS (10, 50, 100, or 200 μmol/L) for 30 min (**D**). Cell viability was measured by the CCK-8 assay (**C**,**E**). ROS levels were determined after pretreated SH-SY5Y cells with L-cysteine (1 mmol/L), SAM (200 μmol/L), or HA (10 mmol/L) before UII (10^−8^ mol/L) treatment (**F**). *n* = 6 in each group. * *p* < 0.05 vs. aCSF group; ^#^ *p* < 0.05 vs. aCSF + UII group.

In vitro, we firstly incubated the SH-SY5Y cells with several concentrations of UII (10^−10^, 10^−9^, 10^−8^, 10^−7^, and 10^−6^ M, Figure 7A) and different exposure times (5, 10, 15, and 20 min, Figure 7B), respectively. Dihydroethidium (DHE) fluorescence intensity results showed that UII significantly increased ROS levels in SH-SY5Y cells at the dose of 10^−8^ mol/L for 10 min. The effect of UII is both concentration- and time-dependent. Preincubating SH-SY5Y cells with UII (10^−8^ mol/L) with various doses of NaHS (10, 50, 100, and 200 μM, Figure 7D) for 5 min before treatment, we found that NaHS exerts its inhibitory effect on UII at the dose of 50 μmol/L. L-cysteine (a substrate for H_2_S, 1 mmol/L) and SAM (a CBS agonist, 200 μmol/L) pretreatment can also significantly inhibit the increase in ROS level caused by UII, while pretreatment with HA (a CBS inhibitor, 10 mmol/L) had no effect on ROS production (Figure 7F). Cell Counting Kit-8 (CCK-8) assay showed that UII (Figure 7C) and NaHS (Figure 7E) treatment had no significant effect on the SH-SY5Y cells viability. These results suggested that both exogenous and endogenous H_2_S could inhibit the increase in ROS levels caused by UII.

### 3.4. Effects of H_2_S on UII-Induced MAPK Activation

**Figure 8:**
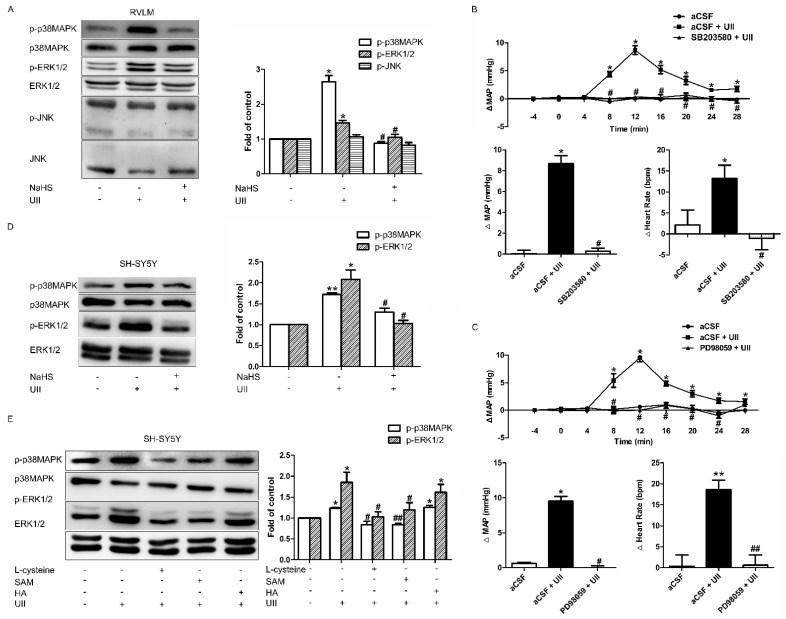
Inhibitory role of H_2_S on UII-induced p38MAPK and ERK1/2 activation. Representative Western blots analysis showing the change in p−ERK1/2, p-p38MAPK, and p−JNK after microinjection of aCSF (*n* = 6), aCSF + UII (*n* = 6), or NaHS + UII (*n* = 6) into the RVLM of SD rats (**A**). Microinjection of PD98059 and SB203580 into the RVLM decreased UII−induced MAP and HR rise in SD rats. Time course of MAP in response to microinjection of aCSF, aCSF + UII, or SB203580 (0.5 μg/0.1 μL, (**B**))/PD98059 (0.1 μg/0.1 μL, (**C**)) + UII. Representative Western blots analysis showing the change in p−ERK1/2 and p−p38MAPK in SH−SY5Y cells after pretreatment with exogenous H_2_S (NaHS, 50 μmol/L) (**D**) or endogenous H_2_S (L−cysteine 1 mmol/L, SAM 200 μmol/L, HA 10 mmol/L) (**E**). *n* = 6 in each group. * *p* < 0.05, ** *p* < 0.01 vs. aCSF group; ^#^ *p* < 0.05, ^##^ *p* < 0.01 vs. aCSF + UII group.

According to the study of Chan et al. [7], MAPK signaling pathway is involved in the Ang-II–induced pressor effect. Thus, we hypothesized that H_2_S might exert its inhibitory role through the MAPK signaling pathway. The results suggest that microinjection of UII (0.5 nmol/0.1 μL) into the RVLM resulted in the activation of p38MAPK or ERK1/2 but not SAPK/JNK. The UII–induced p38MAPK or ERK1/2 phosphorylation was significantly antagonized by coadministration of NaHS (400 pmol/0.1 μL, Figure 8A). Microinjection of SB203580 (0.5 μg/0.1 μL, Figure 8B) or PD98059 (0.1 μg/0.1 μL, Figure 8C), inhibitors of p38MAPK and ERK1/2, respectively, into the RVLM significantly blunted the increase in MAP and HR induced by UII.

As shown in Figure 8, increased phosphorylation of p38MAPK or ERK1/2 caused by UII in SH-SY5Y cells can be significantly decreased by NaHS (50 μmol/L, Figure 8D), L-cysteine (1 mmol/L), or SAM (200 μmol/L, Figure 8E). In contrast, HA (10 mmol/L) had no effect on UII–induced phosphorylation of ERK1/2 or p38MAPK (Figure 8E). Furthermore, none of those treatments elicited a discernible effect on total p38MAPK, ERK1/2, or SAPK/JNK levels in the ventrolateral medulla.

### 3.5. ROS Is in the Upstream of p38MAPK/ERK1/2

**Figure 9:**
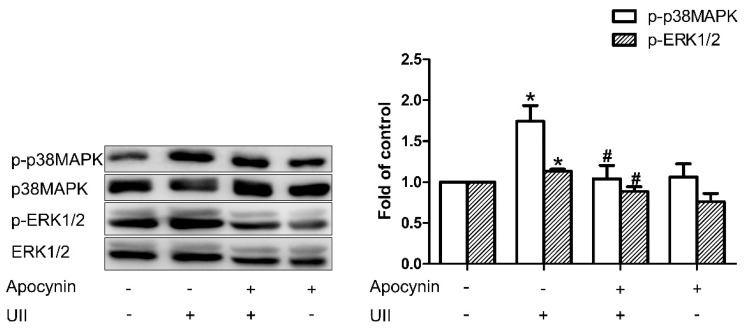
Representative Western blots analysis showing the relationship between ROS and MAPK in SH−SY5Y cells. The change in p−ERK1/2 and p−p38MAPK after pretreated with Apocynin. *n* = 6 in each group. * *p* < 0.05 vs. aCSF group; ^#^ *p* < 0.05 vs. aCSF + UII group.

In exploring whether ROS is in the upstream of p38MAPK/ERK1/2, we detected the change in the phosphorylation of ERK1/2 and p38MAPK in SH-SY5Y cells after preincubation with Apocynin. The results revealed that SH-SY5Y cells pretreated with Apocynin could significantly inhibit the increase in phosphorylation of ERK1/2 and p38MAPK caused by UII (Figure 9). These results suggested that ROS is upstream of p38MAPK/ERK1/2.

### 3.6. Effects of H_2_S on UII-Induced Gα_o_ Activation

**Figure 10:**
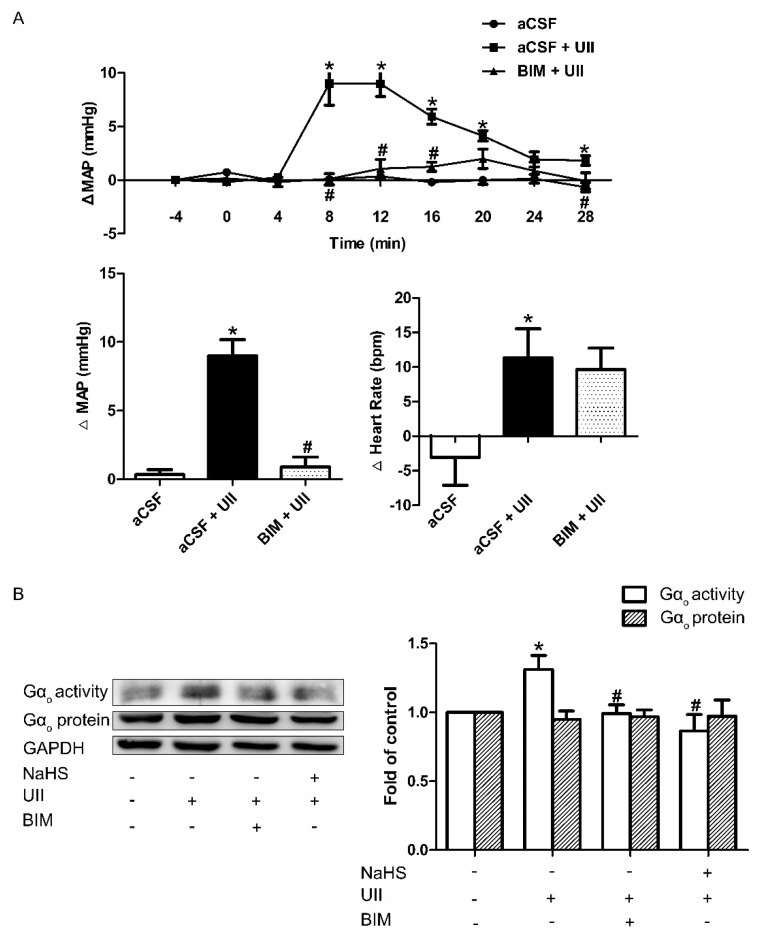
Inhibitory role of NaHS on UII−induced Gα_o_ activation. Time course of MAP and the maximal changes in MAP and HR in response to microinjection of aCSF, aCSF + UII, or BIM + UII into the RVLM of SD rats (**A**). The change in the activity and protein level of Gα_o_ after pretreatment with NaHS or BIM for 30 min before UII was used (**B**). Densitometric analysis of the viability of Gα_o_ (**B**). Mean ± SEM. *n* = 6. * *p* < 0.05 vs. aCSF group; ^#^ *p* < 0.05 vs. aCSF + UII group.

UII specifically binds the UII receptor, which displayed coupling to Gα_o_ and induced ERK1/2 and p38MAPK phosphorylation. As we mentioned above, UT can activate the MAPK signaling pathway coupled to Gα_o_, and ROS is upstream of the MAPK pathway, so we hypothesized that H_2_S might decrease ROS levels through the inhibition of Gα_o_ activity. Firstly, we performed the microinjection in the RVLM area of SD rats. The results showed that pretreatment with microinjection of BIM23127 (BIM, a UII receptor antagonist, 0.2 μg/μL) into the RVLM can significantly attenuate the increase in MAP and HR induced by UII (Figure 10A).

Then Gα_o_ protein expression and its activity were measured in SH-SY5Y cells 10 min after the cells were preincubated with BIM or NaHS and before the treatment of UII. The results showed that coadministration with BIM or NaHS could significantly attenuate the UII-induced Gα_o_ activity. However, the expression of the Gα_o_ protein remained unchanged (Figure 10B).

## 4. Discussion

**Figure 11:**
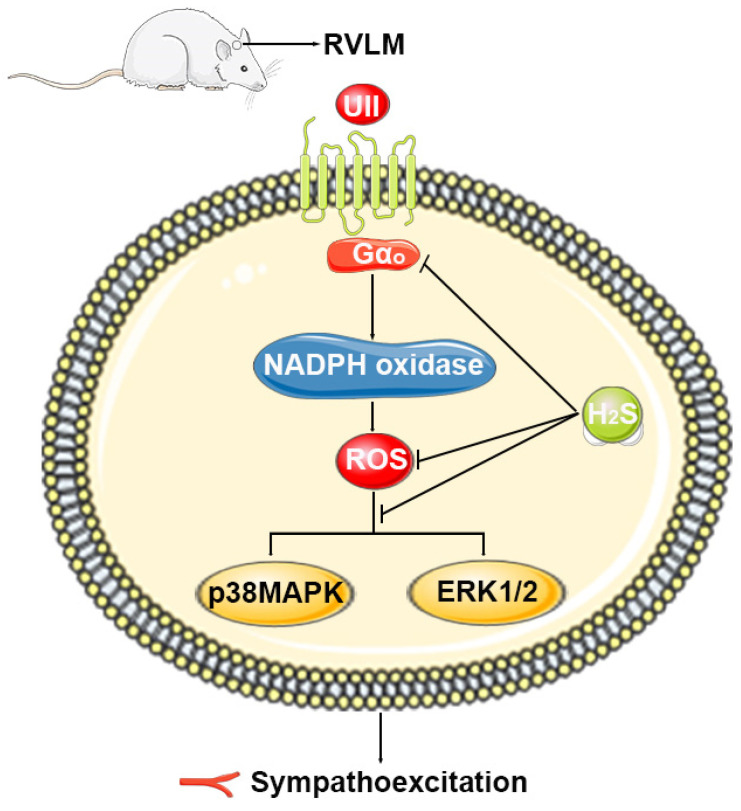
Schematic diagram of the mechanism of H_2_S in UII-induced cardiovascular effects.

It is well established that UII participates in central cardiovascular control through the activation of UT. H_2_S elicits an antihypertensive effect through reduced ROS production. In the present study, we provide the first evidence demonstrating that H_2_S may exert an inhibitory role in UII-induced cardiovascular effects via Gα_o_-ROS-p38MAPK/ERK1/2 pathway. Our statement is supported by the following four findings: (1) The metabolic system of H_2_S was present in the RVLM of SD rat and SH-SY5Y cells. Pretreatment with microinjection of H_2_S into the RVLM significantly attenuated UII-induced MAP and HR rise. (2) H_2_S decreased the UII-induced MAP and HR by inhibiting the activity of NADPH oxidase, phosphorylation of its subunit, p47^phox^, and ROS production. (3) Decreased phosphorylation of p38MAPK and ERK1/2 plays an active role in the inhibitory role of H_2_S on UII-induced cardiovascular effects, and p38MAPK/ERK1/2 pathway is in the downstream of ROS. (4) The activity of Gα_o_ was also involved in the inhibitory role of H_2_S on UII-induced cardiovascular effects (Figure 11).

UII is the most potent vasoactive peptide identified in mammals, which is involved in the pathophysiology of systemic and pulmonary hypertension. Increased UII levels have been reported in patients with hypertension [4] or cardiovascular diseases [15]. Although the peripheral effects of UII are reported to be different, its central effects are invariable in different species [16]. Intracerebroventricular administration of UII in vertebrates provokes hypertension [17]. We previously reported that intracerebroventricular infusion UII significantly increased MAP in SHR rats [6]. However, in different brain regions, UII exerts distinct roles in cardiovascular regulation were reported [18]. In the present study, we found that in the RVLM, a key area for the regulation of arterial blood pressure, MAP was significantly increased by the administration of UII in SD rats (Figure 3). This result is supported by many other studies [16,17]. However, a recent prospective study demonstrated that UII is unlikely to play a role in the development of hypertension [19]. Since it was an observational study, the authors may need to provide more evidence to confirm their conclusions.

As a novel gasotransmitter, evidence highlights a crucial role of H_2_S in numerous diseases, including hypertension, cardiovascular disease, and neurodegenerative diseases. CBS is the major H_2_S-producing enzyme in the CNS. CBS produces H_2_S by using L-cysteine as the main substrate, and its activity is regulated by SAM and HA, agonist and inhibitor of CBS, respectively. In the present study, the expression of CBS was confirmed in the RVLM (Figure 1) and in SH-SY5Y cells (Figure 2), a neuroblastoma cell line. SH-SY5Y cells were identified as glutamatergic neurons (Figure 2).

H_2_S has been reported to protect SHR against hypertension [20]. Treatment with NaHS for 3 months can significantly reduce blood pressure of SHR [21]. Dillon GA et al. firstly found in adults with hypertension that H_2_S-dependent microvascular vasodilation is improved following 16 weeks of sulfhydryl-donating antihypertensive treatment [22]. Furthermore, IP administration of NaHS in a rat model lowered angiotensin II-induced blood pressure rise and prevented the development of hypertension [23]. In agreement with these studies, our results show that pretreatment with microinjection of NaHS (Figure 3), SAM, or L-cysteine (Figure 4) into the RVLM reduced the UII-induced MAP and HR in SD rats. These studies indicate that increase in the expression of both endogenous and exogenous H_2_S may significantly inhibit UII-induced cardiovascular effects. However, the mechanisms that underlie this antihypertensive effect remain largely unknown.

It is worth noting that higher oxidative stress was reported in hypertensive patients [4], and oxidative damage is crucial in the pathogenesis associated with cardiovascular injury in hypertension [24]. In CNS, an imbalance between ROS production and degradation caused by oxidative stress was reported to contribute to the pathophysiology of hypertension [25], elevated O_2_^−^, H_2_O_2_, and baseline ROS in the RVLM, leading to hypertension in SHR [26]. Our current study shows that ROS production and NADPH oxidase activity were also increased by UII (Figure 5). Coadministration with Tempol or Apocynin remarkably inhibited the vasoactive effect caused by UII in the RVLM of SD rats (Figure 6). We also found in SH-SY5Y cells that ROS production significantly increased after the cells were treated with UII (10^−8^ mol/L) for 10 min (Figure 7). These results indicate that ROS play a vital role in UII-induced hypertension.

H_2_S has been proven to be an antihypertensive substrate and reduced ROS production may contribute to its antihypertensive effects [27]. H_2_S also modulates blood pressure via KATP and Kv channels [28]. We reported previously that H_2_S exerts an antihypertensive effect by suppressing ROS production. Microinjection of NaHS, SAM, Apocynin, or Tempol decreased the level of O_2_^−^ in the RVLM of SHR. NADPH oxidase is a major enzyme for superoxide production in the brain. We assessed the activity of NADPH oxidase and found that microinjection of NaHS, SAM, and Apocynin decreased NADPH oxidase activity significantly [2]. In this regard, we pretreated the RVLM or SH-SY5Y cells with NaHS, L-cysteine, or SAM, and the results showed that the increased ROS production induced by UII could be significantly decreased by either exogenous or endogenous H_2_S, while preincubated SH-SY5Y cells with HA had no effect on UII-induced ROS production (Figure 5 and Figure 7). These observations suggest that ROS may be involved in the inhibitory role of H_2_S on UII-induced oxidative stress.

The main source of ROS in CNS is NADPH oxidase [29,30]. Increased ROS generation in the RVLM elevated the MAP and sympathoexcitation of SHR, probably by upregulating NADPH oxidase [31]. NADPH oxidase consists of two membrane-bound gp91^phox^ and p22^phox^ and three cytosolic subunits, such as p47^phox^ and p67^phox,^ as well as small GTPase Rac. Phosphorylation of p47^phox^ is key to the assembly process of the activated NADPH oxidase [32]. Increased enzyme activity of NADPH oxidase was observed in the RVLM of SHR. The enhanced central sympathetic outflow in SHR was blunted when the gene transcription of p47^phox^, gp91^phox^, and p22^phox^ subunits were knocked down in the RVLM [5,33]. The mRNA and protein expressions of the p47^phox^, gp91^phox^, p67^phox^, and p40^phox^ are upregulated in the RVLM of the UII-induced neurogenic hypertension model [7,34,35]. The present study proved that the NADPH oxidase activity was increased in the RVLM of the UII-induced hypertension rat. Pretreatment with microinjection of H_2_S into the RVLM exerted an antihypertensive role in the UII-induced hypertension rat model through decreased phosphorylation of p47^phox^. We observed the same mechanism in SH-SY5Y cells (Figure 5). These results demonstrated that H_2_S in the RVLM is attributable to attenuating UII-induced hypertension by inhibiting the phosphorylation of p47^phox^.

According to the above observed phenomenon, H_2_S could attenuate the enhanced MAP and HR induced by UII via decreased ROS production, while the underlying signaling pathways remain unclear. It has been reported that multiple signaling pathways, including the mitogen-activated protein kinase (MAPK) signaling pathway, are associated with ROS actions in the RVLM. The activities of MAPK (p38MAPK, ERK1/2, and JNK) are strengthened by angiotensin II through an AT1R-dependent ROS production in CNS [7,36,37]. In H_2_O_2_-induced apoptotic cardiomyocytes, UII promotes the phosphorylation of ERK1/2 and increases H_2_S production via upregulating CSE levels [38]. Thus, we speculate that the MAPK signaling pathway may participate in the antihypertensive effect of H_2_S. In the present study, the detection of the phosphorylation of the MAPK family in the RVLM revealed that UII potentiated the activation of p38MAPK and ERK1/2 but not JNK. Pretreated with H_2_S significantly suppressed UII-induced enhanced phosphorylation of p38MAPK and ERK1/2. This result was further confirmed by microinjection of selective p38MAPK or ERK1/2 inhibitor into RVLM. Similarly, we also found in SH-SY5Y cells that H_2_S inhibits UII-induced activation of p38MAPK and ERK1/2 (Figure 8). These results indicatethat ROS and p38MAPK/ERK1/2 pathway are both involved in the inhibitory role of H_2_S on UII-induced cardiovascular effects. However, the upstream–downstream relationship between ROS and p38MAPK/ERK1/2 pathway remains to be explored.

It is generally accepted that ROS production induces the activation of p38MAPK and ERK1/2 [7,39]. However, there are reports that support the opposite opinion [40]. To confirm the upstream–downstream relationship between ROS and p38MAPK/ERK1/2, we used Apocynin and UII in SH-SY5Y cells. We found that NADPH oxidase activity was significantly inhibited by the administration of Apocynin, followed by suppression of UII-induced p38MAPK and ERK1/2 phosphorylation (Figure 9). Our observations suggest that in the underlying signaling pathways of H_2_S in UII-induced cardiovascular effects, ROS may be in upstream of the p38MAPK/ERK1/2 pathway.

As we all know, H_2_S has many cellar and molecular targets. The antihypertensive role of H_2_S in UII-induced cardiovascular effects is highly complex. UII exerts a vasoactive effect through binding to UT, which is primarily coupled to several G proteins, such as Gα_i/o_ and Gα_q/11_. UT coupled to Gα_i/o_, leads to activation of the MAPK signaling pathway. Since ROS is upstream of the MAPK signaling pathway, we hypothesized that H_2_S might decrease ROS levels through the inhibition of Gα_o_ activity. RVLM administration of BIM (UT antagonist, 0.2 μg/0.1 μL) significantly prevented the UII-induced cardiovascular effects. Our results further indicated that UII significantly increased the activity of Gα_o_, while pretreated with H_2_S attenuated the activity of Gα_o_. The protein expression of Gα_o_ remains unchanged in each group (Figure 10). These observations indicate that H_2_S may exert an inhibitory role in UII-induced cardiovascular effects via Gα_o_-ROS-p38MAPK/ERK1/2 pathway, and Gα_o_ might be the potential target of H_2_S.

## 5. Conclusions

In summary, the present study indicated that both endogenous and exogenous H_2_S attenuate UII-induced cardiovascular effects through Gα_o_-ROS-p38MAPK/ERK1/2 pathway. This may provide a novel approach to counteract sympathoexcitation in cardiovascular disorders that are associated with enhanced brain UII. Our study revealed the important value of H_2_S as a new potential therapeutic pathway against hypertension.

## Data Availability

Data is contained within the article.
